# The Comparative Utility of Viromer RED and Lipofectamine for Transient Gene Introduction into Glial Cells

**DOI:** 10.1155/2015/458624

**Published:** 2015-10-11

**Authors:** Sudheendra Rao, Alejo A. Morales, Damien D. Pearse

**Affiliations:** ^1^The Miami Project to Cure Paralysis, University of Miami Miller School of Medicine, Miami, FL 33136, USA; ^2^The Departments of Neurological Surgery, University of Miami Miller School of Medicine, Miami, FL 33136, USA; ^3^The Neuroscience Program, University of Miami Miller School of Medicine, Miami, FL 33136, USA; ^4^The Interdisciplinary Stem Cell Institute, University of Miami Miller School of Medicine, Miami, FL 33136, USA

## Abstract

The introduction of genes into glial cells for mechanistic studies of cell function and as a therapeutic for gene delivery is an expanding field. Though viral vector based systems do exhibit good delivery efficiency and long-term production of the transgene, the need for transient gene expression, broad and rapid gene setup methodologies, and safety concerns regarding *in vivo* application still incentivize research into the use of nonviral gene delivery methods. In the current study, aviral gene delivery vectors based upon cationic lipid (Lipofectamine 3000) lipoplex or polyethylenimine (Viromer RED) polyplex technologies were examined in cell lines and primary glial cells for their transfection efficiencies, gene expression levels, and toxicity. The transfection efficiencies of polyplex and lipoplex agents were found to be comparable in a limited, yet similar, transfection setting, with or without serum across a number of cell types. However, differential effects on cell-specific transgene expression and reduced viability with cargo loaded polyplex were observed. Overall, our data suggests that polyplex technology could perform comparably to the market dominant lipoplex technology in transfecting various cells lines including glial cells but also stress a need for further refinement of polyplex reagents to minimize their effects on cell viability.

## 1. Introduction

Recent studies have challenged our notions on glia : neuron interactions and the role that glia play in normal physiology as well as in the pathology of disease [1–4]. Thus we are at the crossroads of reexamining our understanding of the role of glia in the nervous system. Glial cells play important functions in immune modulation and responses to injury including scarring, axon guidance, and remyelination repair. Therefore, glial cells from both central (astrocytes, oligodendrocytes, and microglia) and peripheral (Schwann cells) nervous systems are emerging as attractive gene therapy targets in a range of neurological disorders and trauma [5, 6]. Genetic manipulation of glia, to modify their expression of specific molecules, can thus significantly alter their molecular and physiological reactions to the environment, providing a tool for better understanding their function under pathological conditions as well as novel therapeutic targets for neuroprotection and neurorepair [7–9]. Though viral delivery systems remain at the forefront of gene therapeutic approaches, safety concerns and costs remain significant issues. Furthermore, the need for fast development times and transient expression paradigms* in vitro* and* in vivo* for gene delivery applications still incentivize research into the use of nonviral gene delivery methods. Nonviral gene delivery methods have improved enormously in recent years and can offer integration-free expression that is becoming more comparable to that of viral vectors under certain experimental conditions [10]. In targeting glial cells, nonviral genetic manipulation has been performed by physical (ballistic labelling, magnetofection), electrical (electroporation), or chemical methods (cationic polymer, cationic lipid, or calcium phosphate) [11–15].

Despite significant research investigation with chemical transfection formulations of cationic lipids (forming lipoplexes) and cationic polymers (polyplexes), a number of limitations remain that have restricted these nonviral delivery systems from reaching their full potential. The road to a perfect chemical transfection reagent involves crossing many hurdles that include the following: (1) capability to load a broad range of cargoes, (2) highly efficient carrier to cargo ratios, (3) consistent efficiency of delivery in any type of cell culture media, including those containing varying amounts of serum, a routinely used cell culture reagent and a common component of the blood, (4) enhanced transfection efficiency for a very low amount of biomolecule used, (5) ability to aid in the efficient survival and timely escape of the biomolecule into the intracellular milieu from transport compartments such as the endocytosis machinery, and (6) capacity to introduce biomolecules to the nucleus, thus providing the ability to target nondividing cells and allow for a faster outcome in dividing cells [16, 17]. All these characteristics need to be improved without causing toxicity or altering cellular biochemical-molecular signatures. Thus, to achieve these goals, chemical methods for cell transfection are being constantly revised and newer transfection reagents are developed to overcome these limitations and advance the field [18].

Cationic lipid-based transfection reagents (lipoplexes) have dominated the field of nonviral gene delivery since 1987 [19]. Cationic polymers (polyplexes) on the other hand have only attracted attention disproportional to their flexibility in design, formulation, and functionality [16, 20]. Polyethylenimine (PEI) is one of the most highly studied cationic polymers since its first use in 1995. To date, in 9 out of 16 clinical studies employing nonviral transfecting agents, some formulation of PEI has been used [8, 20, 21]. Given the limitations of cationic lipid-based technology, such as colloidal stability, cytotoxicity, and their effects on the lipid metabolism of the cell, there is a growing need to optimize cationic polymer technology and other nonviral delivery methods for clinical and HTS applications [14]. However, most of the cationic polymer based methods are heavily endosome centric. Escaping degradation by endosomal acidification is, therefore, an important requirement for efficient biomolecule delivery. Current research on PEI is focused on increasing the buffering capacity of PEI by adding effective endosomal escape [22]. In that direction, Viromer technology has modified the polycationic PEI core by adding hydrophobic and anionic side chains [23]. The synthetic modification on PEI was performed by emulating the influenza virus hemagglutinin, with an alteration in the charge density of Viromer particles to make their surface charge neutral [23]. This modification provides Viromer particles the ability to be endocytosed in the presence of serum and escape effectively from endosomes [23].

In the current investigation we have evaluated the transfection characteristics of Viromer RED, a novel, synthetic, plasmid-specific carrier molecule based on alkylated and carboxyalkylated branched PEI (Lipocalyx GmbH, Halle (Saale), Germany). Qualitative and quantitative experiments were performed using standard cell culture conditions with a number of cell lines and primary rat glia cell types, comparing Viromer RED to Lipofectamine 3000, a leading cationic lipid-based chemical method of transfection.

## 2. Materials and Methods

### 2.1. Reagents

DMEM-high glucose with pyruvate (11995-065; used for HEK293 and DI TNC1), DMEM-high glucose without pyruvate (11965-092; used for Schwann cells), FBS (16000-044; used for HEK293, DI TNC1 and BV2 after heat inactivation), Penicillin/Streptomycin (15140-122), Trypsin-EDTA (15400-054), HBSS (14170-112), DNAase-RNAase free water (14170-112), DPBS (14190-250), and astrocyte medium (A1261301) were purchased from Gibco, Life Technologies (Carlsbad, CA). Forskolin (F6886), poly-L-lysine (P2636), and other chemical reagents were purchased from Sigma-Aldrich (St. Louis, MO). Heregulin (100-03) was purchased from PeproTech (Rocky Hill, NJ) and pituitary extract (BT-215) was purchased from Biomedical Technologies (Alfa Aeser, Ward Hill, MA). The pMaxGFP plasmid vector for control transfections was obtained from Lonza (Allendale, NJ) and pGL4.13 (E668A) was procured from Promega (Madison, WI). The MidiPlus kit for plasmid preparation was purchased from Qiagen (Valencia, CA). Lipofectamine 3000 was purchased from Life Technologies (Carlsbad, CA). Viromer RED was procured from Lipocalyx, Germany. Costar 96-well assay plate for luminometry (3610) was purchased from Corning Inc. (Corning, NY). CellTiter-Fluor cell viability assay (G6080), the Luciferase Assay System (E4030), and 5X passive lysis buffer (E1941) were purchased from Promega (Madison, WI). Anti-GFAP antibody (Z0334) was purchased from Dako (Carpinteria, CA) and anti-S100 antibody (S-2532) was purchased from Sigma (St. Louis, MO). Goat anti-mouse and goat anti-rabbit Alexa Fluor 594 were purchased from Life Technologies (Carlsbad, CA) and Hoechst 33342 (H3570) was purchased from Invitrogen, Molecular Probes (Life Technologies (Carlsbad, CA)).

### 2.2. Animals

Adult female Fischer rats (as Schwann cell donors; Harlan Laboratories, Indianapolis, IN) and adult pregnant female Lewis rats (with E18-19 pups as cortical astrocyte donors; Charles River Laboratories, Wilmington, MA) were housed in accordance with National Institutes of Health Guidelines and the Guide for the Care and Use of Laboratory Animals. The Institutional Animal Care and Use Committee of the University of Miami approved all animal procedures. Efforts were made to minimize the number of animals used and to decrease animal suffering. Adequate anesthesia (70 mg/kg ketamine, 5 mg/kg xylazine) was determined by monitoring the corneal reflex and hindlimb withdrawal to painful stimuli. During surgery (sciatic nerve harvest for Schwann cell culture), the rats were kept on a heating pad to maintain body temperature at 37 ± 0.5°C. Pregnant Lewis rats were euthanized with CO_2_ and decapitated before retrieving the pups for harvesting cortices. Rats were housed two per cage at a temperature of 24°C, 12 hr dark/light cycle with* ad libitum* access to water and food.

### 2.3. Cell Culture

HEK293 (ATCC CRL-1573) and DI TNC1 (ATCC CRL-2005) and BV2 cell lines were cultured in D10 (DMEM-10% FBS with 1X Pen/Strep). Schwann cells harvested from adult rat sciatic nerves [24] were seeded on poly-L-lysine (PLL) coated petri dishes in D10-3M media (D10, 2 *μ*M Forskolin, 10 nM Heregulin, and 20 *μ*g/mL of pituitary extract). Rat astrocytes isolated from E18-19 rat cortices [25] were cultured in astrocyte medium (A1261301, Gibco, Carlsbad, CA) and grown in 75 cm^2^ vented culture flasks. All cell cultures were maintained inside a humidified incubator at 37°C and 5% CO_2_.

### 2.4. Transfection

Cells were seeded on either 24- or 96-well cell culture treated plates at a density to ensure ~80% confluent cultures at 24 hr after seeding. Typically 80,000 cells/cm^2^ surface area of the culture dish were seeded in cell-specific medium. Transfection using Lipofectamine 3000 was performed according to the manufacturer's protocol with a DNA to Lipofectamine ratio of 1 : 3 w/v. A transfection enhancer, the 3000 enhancer reagent (1 : 2, DNA : Reagent, w/v), was used along with the Lipofectamine 3000 transfection reagent for all transfections. Typically 100 ng and 500 ng of plasmid DNA were transferred to each well of the 96-well plate and 24-well plates, respectively. The standard complexation protocol was employed for Viromer RED according to the manufacturer's instructions. Briefly, Viromer RED was diluted (1 : 24 v/v) and the plasmid DNA was diluted independently to 18 ng/*μ*L in the provided dilution buffer E. A 22 *μ*L volume of diluted DNA was added to 4 *μ*L of diluted Viromer RED and the mixture then was allowed to stay at room temperature for 15 min. For transfection, 100 ng of plasmid DNA, Viromer RED mixture, was added to each well of the 96-well plate by dispensing 6.7 *μ*L per well. In specified experiments, 6 hr before transfection, the media was changed to either DMEM with antibiotics and without serum or DMEM with antibiotics and with serum. For quantitative evaluation of protease activity (as a measure of cell viability), luminometry, or fluorescence microscopy, cells were used 24 hr after transfection.

### 2.5. Viability Assay and Multiplexing Luminometry

The viability assay was performed using the CellTiter-Fluor kit (Promega). Briefly, 80,000 cells/cm^2^ were seeded on a 96-well plate and transfected with 100 ng/well of pGL4.13 plasmid using either Lipofectamine 3000 or Viromer RED. For Schwann cells, the 96-well plate was precoated using PLL for at least one hour. At 24 hr after transfection, 20 *μ*L of 5X assay reagent (containing GF-AFC; glycyl-phenylalanyl-aminofluorocoumarin-a fluorogenic cell permeable peptide substrate) was added to the culture wells and incubated at 37°C for 30 min with occasional agitation on the orbital shaker. Plates with assay reagent were protected from ambient light by covering them with aluminum foil. Fluorescence (in relative fluorescence units; RFU) was measured using a fluorometer (SpectraMax M5) with 380 nm (excitation) and 505 nm (emission). Values were normalized to the mean of the nontransfected control for each category and reported as fold change. After RFU measurements, cells were lysed and processed for the luciferase assay.

### 2.6. Luciferase Assay

Cells were seeded at 80,000 cells/cm^2^ density and transfected with 100 ng/well of pGL4.13 luciferase plasmid using either Lipofectamine 3000 or Viromer RED as described above. Nontransfected cells served as a negative control. At 24 hr after transfection, following viability estimation using the CellTiter-Fluor kit (Promega) as described above, the media was aspirated, the wells were washed with DPBS (pH 7.4) and 1X passive lysis buffer (Promega) was directly added to the wells for incubation at room temperature for 15 minutes to lyse the cells. Typically, 20 *μ*L of 1X passive lysis buffer (reconstituted in water) was added to each well of the 96-well plate and subjected to brief agitation on an orbital shaker. After cell lysis, 100 uL of luciferase assay reagent (Promega) was added to each well and the measurement of luminescence was performed using a microplate reader (SpectraMax M5) at 37°C with 1,500 milliseconds of integration time. Readings were taken at least twice and averaged. Average luminescence (in relative light units; RLU) was divided by the mean RFU obtained for the same well from using the CellTiter-Fluor kit (Promega). Resultant values were further normalized to the mean RLU/RFU ratio of the nontransfected control for each category and reported as a fold change.

### 2.7. Immunofluorescence

Cells were fixed with 4% paraformaldehyde and washed with phosphate-buffered saline (PBS). Following permeabilization with 0.2% Triton X-100 in PBS for 10 min and 30 min of blocking with 5% BSA in PBS, they were incubated overnight with anti-S100 (1 : 200) or -GFAP (1 : 1,000) primary antibodies in blocking buffer. Subsequently, they were given three washes with PBS and were incubated for 45 min with secondary antibody solution containing goat anti-rabbit Alexa Fluor 594 (1 : 500) or goat anti-mouse Alexa Fluor 594 (1 : 500) diluted in 5% BSA + 5% heat-inactivated goat serum in PBS. Hoechst was added at 1 : 1,000 dilution to the secondary antibody solution. After incubation, cells were washed with PBS and kept in PBS until imaging.

### 2.8. Imaging

Imaging of cultures under bright-field and fluorescence microscopy was performed using an inverted fluorescence microscope (Olympus IX70, Center Valley, PA and Zeiss apo tome Thornwood, NY) with 10X, 20X objectives, DIC Nomarski, EGFP, Cy3, and DAPI filters. Images acquired on the Olympus microscope were saved in TIFF format while for the Zeiss Apo tome the ZVI format was used. ImageJ was employed to extract individual channels from the ZVI format and resave them in TIFF format. After processing involving multichannel generation, brightness-contrast (brightness +50–150, contrast 10–100), level adjustments (0–80, 1.4–4, 255), montage, and channel mixing were performed using AdobePhotoshop CS6 (64 bit, San Jose, CA) with post-processing consistent across comparative images.

### 2.9. Data Analysis and Statistics

All quantitative experiments were performed in quadruplicates. Results were tabulated in Microsoft Excel and statistical tests and graph generation was carried out in SPSS v.22. At least 400 cells were counted for each condition. The number of transfected cells were reported as mean ± SD. Comparisons were typically made either between transfecting reagent groups and/or to control groups that included untreated or transfected cells without DNA cargo. ANOVA with multiple comparisons followed by a Bonferroni* post hoc* test was used to compute *P* values. In other conditions, a two-tailed Student's *t*-test with the assumption of equal variance was used to compare two samples. A *P* value of < 0.05 was defined as statistically significant.

## 3. Results

### 3.1. Viromer RED Shows Comparable Transfection to Lipofectamine 3000 in a Diversity of Cell Lines

**Figure 1:**
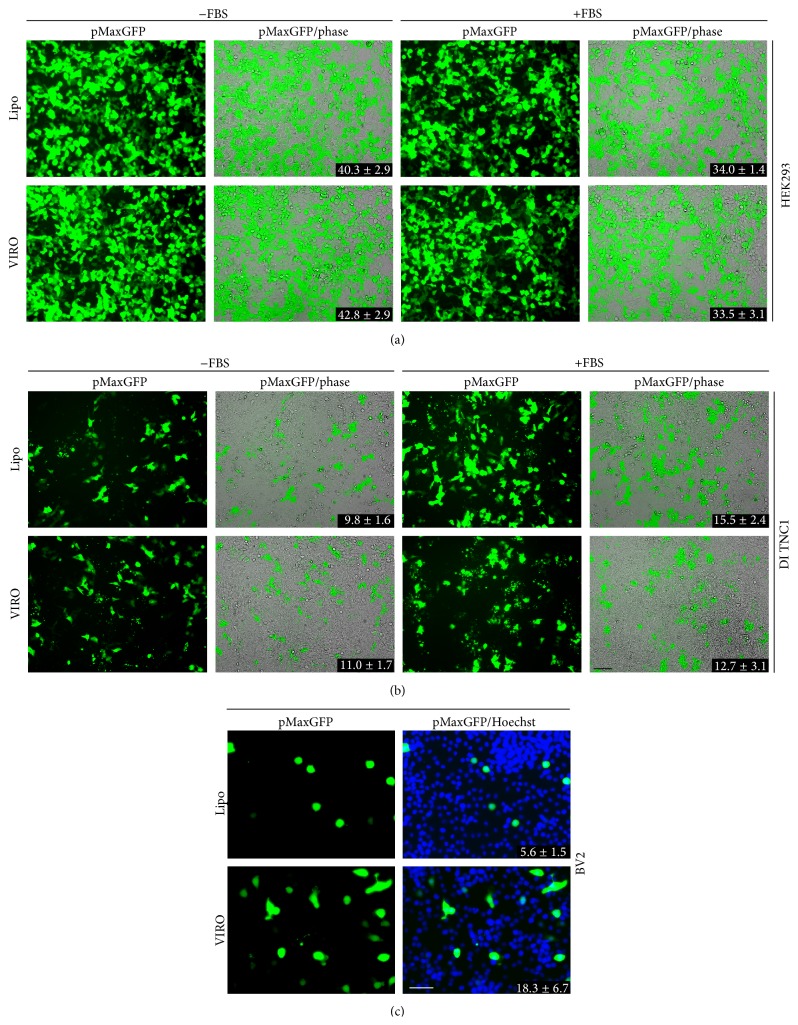
Viromer RED shows comparable transfection to Lipofectamine 3000 in a diversity of cell lines: HEK293, DI TNC1, and BV2 cells were transfected with pMaxGFP using Viromer RED (VIRO) or Lipofectamine 3000 with enhancer (Lipo). Transfections were performed either in serum (+FBS) or without serum (−FBS). Serum containing media was added to the cells 4–6 hr after transfection. Phase contrast and fluorescence images show a robust transfection efficiency by both reagents in HEK293 (a) that decreases in the presence of serum (a, right). The transfection efficiency was lower in the DI TNC1 cell line compared to HEK293 (b). Viromer RED transfected a higher percentage of BV2 cells with pMaxGFP vector (c) as compared to Lipofectamine 3000 with enhancer. Numbers represent percentage mean ± SD transfected cells.

Human embryonic kidney (HEK293) and immortalized rat diencephalon astrocyte (DI TNC1) cell lines were transfected in the presence or absence of serum with a pMaxGFP plasmid using either Lipofectamine 3000 or Viromer RED and 6 hr after transfection the media changed to one that either had serum or did not. Mouse BV2 microglial cells were transfected similarly but in the presence of serum. In the case of BV2, media was changed to one containing serum 4 hr after transfection. Fluorescence images were captured 24 hr after the transfection and the semiquantitative analysis of images (Figure 1(a)) suggested that both Lipofectamine 3000 and Viromer RED had a comparable transfection capability on HEK293 in the absence of the serum (%; Lipo-NoFBS 40.3 ± 2.9; VIRO-NoFBS 42.8 ± 2.9), which decreased after addition of serum (%; Lipo-FBS 34 ± 1.4; VIRO-FBS 33.5 ± 3.1) (Figure 1(a)). The transfection efficiency of these agents on the DI TNC1 cell line was lower than that of HEK293, though there was minimal reduction in transfection rate after addition of serum (%; Lipo-NoFBS 9.8 ± 1.6; VIRO-NoFBS 11.0 ± 1.7; Lipo-FBS 15.5 ± 2.4; VIRO-FBS 12.7 ± 3.1) (Figure 1(b)). In contrast, Viromer RED appeared to transfect at a higher rate in the microglial cell line, BV2 (%; 18.3 ± 6.7), as compared to Lipofectamine 3000 (5.6 ± 1.5, *P* = 0.0324) (Figure 1(c)).

### 3.2. Viromer RED Shows Comparable Transfection to Lipofectamine 3000 in Primary Glial Cells

**Figure 2:**
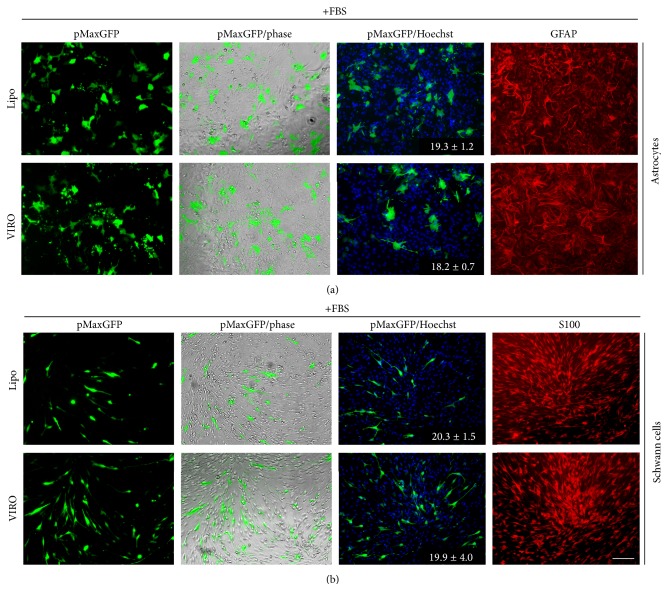
Viromer RED shows comparable transfection to Lipofectamine 3000 in primary glia. Primary rat astrocytes and Schwann cells were transfected with pMaxGFP using Viromer RED (VIRO) or Lipofectamine 3000 with enhancer (Lipo). Transfections were performed in serum (+FBS). Phase contrast and fluorescence images show a comparable transfection efficiency by both reagents in primary rat astrocytes (a) and primary rat Schwann cells (b). No gross change in the morphology of astrocytes or Schwann cells was observed after transfection using the reagents. Purity of the cultures was assessed using GFAP (for astrocytes, a) and S100 (for Schwann cells, b). Numbers represent percentage mean ± SD transfected cells.

Late embryonic primary rat astrocytes and adult rat Schwann cells were transfected 24 hr after seeding in the presence of serum with a pMaxGFP plasmid using either Lipofectamine 3000 or Viromer RED. Bright-field and fluorescence images were captured 24 hr after the transfection. Anti-GFAP and anti-S100 antibodies were used to demonstrate the purity of the astrocyte and Schwann cell cultures, respectively (purity > 90%). Both Lipofectamine 3000 and Viromer RED had comparable transfection efficiency in both astrocytes (%; Lipo-FBS 19.3 ± 1.2, VIRO-FBS 18.2 ± 0.7, Figure 2(a)) and Schwann cell cultures (%; Lipo-FBS 20.3 ± 1.5, VIRO-FBS 19.9 ± 4.0, Figure 2(b)).

### 3.3. Viromer RED Transfected Cells Exhibit Reduced Viability When Compared to Lipofectamine 3000 Transfected Counterparts

HEK293 cells, primary rat astrocytes, and Schwann cells as well as mouse BV2 microglial cells were transfected with a luciferase producing vector (pGL4.13) using either Lipofectamine 3000 or Viromer RED. At 24 hr after transfection, cells were incubated with 5X Gly-Phe-AFC (GLY-Phe-7-amino-4-trifluoromethylcoumarin); a cell permeable, fluorogenic substrate that is target of dipeptidyl peptidases (in particular Cathepsin C) and is a conserved class of proteases in mammalian cells. Dipeptidyl peptidases cleave the substrate and release the fluorescent substance over 30 min of incubation. Since only live cells can cleave the substrate, relative fluorescence units (RFU) directly correlate with the number of viable cells.

**Figure 3:**
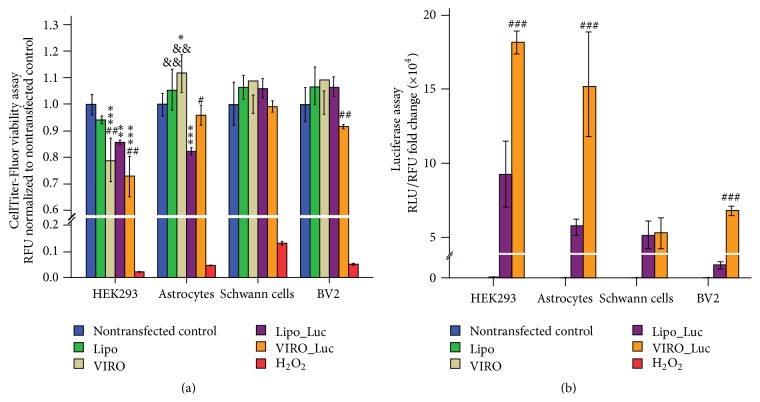
(a) Viromer RED shows differential viability in cell lines and primary cells: HEK293, primary rat astrocytes, primary rat Schwann cells, and BV2 microglial cell were treated with transfection reagents with or without cargo (blue = untreated control, green = Lipofectamine 3000 with enhancer, gray = Viromer RED, purple = Lipofectamine 3000 with enhancer with pGL4.13 luciferase plasmid, orange = Viromer RED with pGL4.13 luciferase plasmid, and red = 1% hydrogen peroxide (H_2_O_2_) treated cells for 5 min before the addition of CellTiter-Fluor reagent). Cells were assessed for viability 24 hr after transfection using CellTiter-Fluor reagent. Absolute fluorescence (RFU measured at 380 nm Excitation/505 nm Emission) was normalized to mean fluorescence of nontransfected cells and fold change reported. Comparisons were made within the untreated control (*∗*), within the transfection reagents (with or without cargo), (&) and across the transfection reagents (within cargo or no cargo group) (#) in the respective cells/cell lines. Values represent mean ± 1 SD. ^*∗*, &, #^ *P* < 0.05; ^*∗∗*, &&, ##^ *P* < 0.01; ^*∗∗∗*, &&&, ###^ *P* < 0.001. (b) Viromer RED transfected cells show a higher luminescence as compared to those transfected with Lipofectamine 3000: HEK293, primary rat astrocytes, primary rat Schwann cells, and BV2 microglial cell lines were treated with transfection reagents, with or without cargo (blue = untreated control, green = Lipofectamine 3000 with enhancer, gray = Viromer RED, purple = Lipofectamine 3000 with enhancer with pGL4.13 luciferase plasmid, and orange = Viromer RED with pGL4.13 luciferase plasmid). Cells were lysed 24 hr after transfection following the assessment of viability (absolute fluorescence (RFU)) using CellTiter-Fluor reagent. Luminescence (RLU) was measured using the luciferase assay reagent. The RLU/RFU ratio data was normalized to the mean RLU/RFU ratio of the nontransfected cells. Comparisons were made across the transfection reagents (with cargo group) (#) in the respective cells/cell lines. Values represent mean ± 1 SD. ^###^ *P* ≤ 0.001.

Fluorometric data (Figure 3(a)) showed differential effects on cell viability when transfection was performed with Viromer RED. Furthermore, the presence or absence of cargo and the cell type being transfected were two parameters that were associated with a change in cell viability after addition of the transfection agents. The greatest disparity in cell viability among the two transfecting agents was observed in HEK293 cells where Viromer RED decreased the cell viability by ~21% (*P* < 0.001; nontransfected control) and the addition of cargo (plasmid pGL4.13) further decreased the viability by ~9% (total decrease ~30%, *P* < 0.001; nontransfected control), whereas addition of cargo to Lipofectamine 3000 decreased HEK293 viability by ~15% (*P* < 0.001; nontransfected control). However, for both Viromer RED and Lipofectamine 3000 there was no significant difference in viability when comparisons were made to the vehicle only controls (*P* = 0.3435, *P* = 0.3461, resp.). In contrast, Viromer RED alone caused a statistically significant ~15% decrease in HEK293 cell viability when compared directly to Lipofectamine 3000 transfected cells (*P* = 0.0063). This difference in viability was maintained when both reagents were loaded with cargos (*P* = 0.00625).

Fluorometric data (Figure 3(a)) for primary rat astrocyte cultures showed no decrease in cell viability with either Lipofectamine 3000 or Viromer RED alone. However, the addition of cargo to Lipofectamine 3000 decreased the viability by ~18% (*P* = 0.0094; nontransfected control, *P* < 0.001; vehicle only control). Fluorometric data (Figure 3(a)) for primary rat Schwann cell cultures did not show any difference in viability after vehicle only or vehicle with cargo transfections when performed using either Viromer RED or Lipofectamine 3000. It is important to note that transfections using both of these reagents along with pMaxGFP plasmid (Figure 3(a)) show similar cargo delivery in Schwann cells. In contrast, cargo loaded Viromer RED was seen to produce ~9% drop in viability as compared to cargo loaded Lipofectamine 3000 (*P* = 0.0090); however, this drop was not significant when compared to either nontransfected or Viromer RED only control (*P* = 0.4383, *P* = 0.2987, resp.).

### 3.4. Viromer RED Transfected Cells Show a Higher Luminescence as Compared to Those Transfected with Lipofectamine 3000

HEK293, primary rat astrocytes, Schwann cells, and mouse BV2 microglial cells were transfected with luciferase vector (pGL4.13) using either Lipofectamine 3000 or Viromer RED and were taken up for multiplexed-luminometry 24 hr after transfection. Media was aspirated and the cells were washed with PBS before lysis and proceeding to luminometry. RLU was divided by the RFU obtained from the same well and normalized to the mean RLU/RFU ratio of the nontransfected control. Transfections in HEK293, primary rat astrocytes, and mouse BV2 microglial cells performed using Viromer RED consistently showed a higher normalized RLU/RFU ratio when compared with the same cells transfected using Lipofectamine 3000 (Figure 3(b); *P* < 0.001), whereas normalized luminescence readings after transfection of luciferase vector using Viromer RED in Schwann cells were not statistically significant compared to Lipofectamine 3000. In addition, we also performed an isolated luminometry experiment wherein we lysed the control, vehicle treated or cargo charged vehicle treated cells with passive lysis buffer and measured the luciferase activity 24 hr after transfection. We observed an upward shift of the highest luminescence but the differences between transfections performed using Viromer RED and Lipofectamine 3000 remained statistically significant (data not shown).

## 4. Discussion

Introduction of exogenous genetic material into glial cells is a powerful technique that provides the capability to manipulate and understand glial cell behavior under various experimental conditions. With the advent of CRISPR/CAS genome editing, HTS, and the optimized drug discovery work flow, the demand for effective, scalable, quick to set up gene delivery methods is an expanding need. Research studies into developing novel nonviral gene delivery agents continue to grow and have provided important data on their feasibility and applicability [26, 27]. This is illustrated by the recent investigation of at least 7 different nonviral delivery systems in various phases of clinical study [18].

In the current study the efficiency of Viromer particles to deliver plasmid DNA to relatively difficult to transfect primary cells (astrocytes and Schwann cells) was tested, following initial evaluation in commonly used cell lines: HEK293, microglial cells (BV2), and rat astrocytes (DI TNC1). For comparison, the latest and leading cationic lipid-based reagent, Lipofectamine 3000 with enhancer [28], was employed. A robust transfection of pMaxGFP plasmid in HEK293 cells was observed which was dramatically lower in DI TNC1 and BV2 cells. A comparable reduction in transfection efficiency was then seen in the presence of serum. However, Viromer RED transfected a higher number of BV2 microglia (*P* = 0.0324) in the presence of serum as compared to Lipofectamine 3000 (with enhancer). Next, the capability of Viromer RED to deliver a luciferase construct to primary cells (astrocyte and Schwann cells) was evaluated and quantified using luminescence 24 hr after transfection. When compared to Lipofectamine 3000, results suggested that luciferase activity was higher in cells transfected using Viromer RED (*P* < 0.001). However, both transfection reagents performed similarly, but poorly, in their ability to transfect Schwann cells. Previously, it has been shown that the transfection of primary rat astrocytes using PEI produces rapid transgene expression when compared to transfection with cationic lipids [29]. In the same investigation it was reported that the cationic lipid-based method displayed a greater plasmid DNA delivery and produced a higher percent transfection with EGFP compared to PEI [29]. In the present study, Lipofectamine 3000 (with enhancer) transfected (pMaxGFP) a higher percentage of cells compared to Viromer RED (Figures 1 and 2). However, when transgene expression was measured through the transfection of a luciferase construct, Viromer RED outperformed Lipofectamine 3000 (with enhancer) (Figure 3(b), *P* < 0.001). This paradox is supported by previous mechanistic studies showing that liposomes hindered the effective transition of DNA complexes from the cytoplasm to nucleus, likely by prolonging their binding to the plasmid DNA [29, 30].

Although Viromer RED technology was specifically designed to overcome the limitations observed with serum on transfection efficiency, in the current investigation both Lipofectamine 3000 (with enhancer) and Viromer RED performed similarly in the presence of serum and in some cases the former appeared superior. However, the addition of serum did exhibit a dramatic effect on pMaxGFP expression when either of the transfection reagents was used (Figure 1). The presence of serum has been well documented to modulate the transfection efficiency of lipoplexes and polyplexes, where in the size of lipoplexes, surface charge density, colloidal stability, and altered uptake mechanisms (caveolae or clathrin dependent) have been suggested to play an important role [31–35]. The current experiments were conducted in 10% FBS and a standard protocol for Viromer preparation was used. The neutral surface charge of Viromer has been suggested to lead to less aggregation in the presence of serum, thus enhancing its functionality [23, 36]. Our results suggest that this enhanced functionality in the presence of serum might be a cell-specific effect and that further optimization of the Viromer design may be necessary to enhance its serum tolerance and reduce the cell to cell variability in transfection efficiency.

Schwann cell transfection by nonviral methods has been attempted before [37, 38]. In one study, the efficiency using FuGENE HD was 2% and with Amaxa© nucleofection was 10% [39]. Another method, employing specialized media and electroporation pushed transfection efficiency to 20–40% [40]. Even though nucleofection remains the method of choice for obtaining high transfection efficiencies, concerns have been raised about the posttransfection aggregation behavior of Schwann cells [39]. In the current study, Schwann cells were subjected to both regular transfection and retrotransfection using Lipofectamine 3000 and Viromer RED. We observed comparable, but low, transfection efficiency with both agents, though we did not observe any change in morphology after transfection (Figure 2(b)). Previously, another study reported no gross changes in cell morphology after transfecting Schwann cells with Lipofectamine 2000, the previous generation cationic lipid-based transfection agent [16]. These results suggest that the observed morphology changes in Schwann cells after electroporation were related to the specific media formulations employed and/or the electroporation parameters used. Our findings regarding Viromer RED and Lipofectamine 3000 (with enhancer agent) compatibility to retrotransfection provide feasibility data in support of research studies interested in exploring attachment characteristics of Schwann cells.

The comparative cell viability among transfecting agents was evaluated using Gly-Phe-AFC, a highly specific cell permeable fluorogenic substrate for the conserved protein Cathepsin C (dipeptidyl aminopeptidase I) [41]. The Gly-Phe-AFC based aminopeptidase assay performs comparably with the ATP assay and is capable of detecting as low as 10 cells per well with generated fluorescence proportional to the number of cells, thus being a marker for cell viability [41]. When equal numbers of cells were seeded and transfected with pGL4.13 luciferase vector using either Lipofectamine 3000 (with enhancer agent) or Viromer RED followed by incubation with Gly-Phe-AFC 24 hr later, Viromer RED alone was shown to decrease the viability of HEK293 cells. The loading of cargo on Viromer RED further decreased the viability of both HEK293 cells and astrocytes (*P* < 0.01), though Schwann cell viability was unaffected. This is probably due to decreased efficiency of transfection in Schwann cells. Cargo induced decreases in viability were also observed for Lipofectamine 3000 (with enhancer agent), though no significant difference between agent and agent with cargo was observed in astrocytes. A marked difference in Cathepsin C activity between HEK293, astrocytes, and Schwann cells was also observed across similar cell numbers. This could be the result of differences in cell cycle times, cell size, and the basal levels of Cathepsin C in the cell lines used.

Nonviral gene delivery agents are constantly redesigned to enhance transfection efficiency and decrease cytotoxicity. PEI has been previously reported to enhance transfection as well as display toxicity depending on its size and concentration, with active endeavors in its modification to improve its cytotoxicity profile [42–44]. Our results suggest that even though Viromer RED provides enhanced transgene expression, it still suffers from toxicity in selected cell lines after cargo loading, though a degree of toxicity was also shared with the leading cationic lipid compound that was comparatively used. Further optimization of cell seeding densities or complexation parameters for different cell systems may provide a way to reduce these negative effects on cell viability [45, 46]. Viromer technology has been further developed to suit the needs of different biomolecules (plasmid DNA, siRNA) and has been investigated previously on both neuronal (SHSY5Y) and nonneuronal cell lines (Ramos, RAW264.7 macrophages) [47–51].

Our findings show that Viromer RED shows substantial transfection efficiency, even in the presence of the serum and is able to transfect a variety of cell lines as well as primary rat glial cells, including astrocytes and Schwann cells. This is the first report to test the capability of the plasmid-specific synthetic transfection agent Viromer RED in comparison to an efficient cationic lipid-based formulation across multiple cell types. The results demonstrate the maturation of PEI based synthetic nonviral delivery vector technology and shows the feasibility of Viromer RED as suitable alternative to leading cationic lipid-based reagents in studies involving regular transfection and high-throughput experiments on primary glial cells.

## 5. Conclusion

In summary, we have evaluated the capability of Viromer RED in transfecting two different plasmid DNAs (fluorescent protein and luciferase encoding) into various cell types, including primary rat glial cells, when compared to an efficient cationic lipid-based transfecting reagent (with enhancer), Lipofectamine 3000. We observed their comparable capability to transfect immortalized cells and primary astrocytes in the presence of the serum. We also observed an enhanced transgene activity using the luciferase system in the presence of Viromer RED. Overall the current study shows that with additional optimization of its cytotoxicity profile, especially after cargo loading, and with suitable functionality grafting, Viromer technology can position itself as a suitable alternative to prevalent cationic lipid technology. Our study concludes that Viromer RED is retrotransfection compatible in rat astrocytes and Schwann cells and, therefore, with proper optimization can be used for studies dealing with cell attachment and HTS based applications.
